# Impaired arbitration between reward-related decision-making strategies in Alcohol Users compared to Alcohol Non-Users: a computational modeling study

**DOI:** 10.1038/s44277-024-00023-8

**Published:** 2025-01-03

**Authors:** Srinivasan A. Ramakrishnan, Riaz B. Shaik, Tamizharasan Kanagamani, Gopi Neppala, Jeffrey Chen, Vincenzo G. Fiore, Christopher J. Hammond, Shankar Srinivasan, Iliyan Ivanov, V. Srinivasa Chakravarthy, Wouter Kool, Muhammad A. Parvaz

**Affiliations:** 1 Department of Health Informatics, Rutgers - School of Health Professions, Piscataway, NJ USA; 2 Department of Psychiatry, Icahn School of Medicine at Mount Sinai, New York, NY USA; 3 Department of Biotechnology, Indian Institute of Technology, Madras, Chennai India; 4 University of Pittsburgh School of Medicine, Pittsburgh, PA USA; 5 Department of Psychiatry & Behavioral Sciences, Division of Child & Adolescent Psychiatry, Johns Hopkins University School of Medicine, Baltimore, MD USA; 6 Department of Psychological & Brain Sciences, Washington University in St. Louis, St. Louis, MO USA; 7 Department of Neuroscience, Icahn School of Medicine at Mount Sinai, New York, NY USA; 8 Department of Artificial Intelligence and Human Health, Icahn School of Medicine at Mount Sinai, New York, NY USA

**Keywords:** Human behaviour, Cognitive neuroscience

## Abstract

Reinforcement learning studies propose that decision-making is guided by a tradeoff between computationally cheaper model-free (habitual) control and costly model-based (goal-directed) control. Greater model-based control is typically used under highly rewarding conditions to minimize risk and maximize gain. Although prior studies have shown impairments in sensitivity to reward value in individuals with frequent alcohol use, it is unclear how these individuals arbitrate between model-free and model-based control based on the magnitude of reward incentives. In this study, 81 individuals (47 frequent Alcohol Users and 34 Alcohol Non-Users) performed a modified 2-step learning task where stakes were sometimes high, and other times they were low. Maximum *a posteriori* fitting of a dual-system reinforcement-learning model was used to assess the degree of model-based control, and a utility model was used to assess risk sensitivity for the low- and high-stakes trials separately. As expected, Alcohol Non-Users showed significantly higher model-based control in higher compared to lower reward conditions, whereas no such difference between the two conditions was observed for the Alcohol Users. Additionally, both groups were significantly less risk-averse in higher compared to lower reward conditions. However, Alcohol Users were significantly less risk-averse compared to Alcohol Non-Users in the higher reward condition. Lastly, greater model-based control was associated with a less risk-sensitive approach in Alcohol Users. Taken together, these results suggest that frequent Alcohol Users may have impaired metacontrol, making them less flexible to varying monetary rewards and more prone to risky decision-making, especially when the stakes are high.

## Lay Summary

This study explored decision-making in frequent alcohol users versus alcohol non-users. Normally, people use more thoughtful strategies when rewards are high. The study shows alcohol users are not doing so and remain less cautious and take more risks when stakes are high. This suggests that frequent alcohol users may struggle to adapt their decision-making based on reward size, leading to riskier choices especially when potential gains are significant.

## Introduction

Excessive alcohol consumption and alcohol use disorders (AUD) are growing public health concerns, costing $249 billion annually in the United States alone [1, 2]. During the COVID-19 pandemic, deaths from excessive alcohol and AUD increased, especially among women [3–5]. Therefore, it is vital to increase our understanding of neurobehavioral factors linked to alcohol consumption and its consequences. Impaired decision-making is one such behavioral aspect that is found to be associated with alcohol and substance use disorders. Research in this domain has painted the relatively straightforward picture that substance abuse reduces reward sensitivity [6–11], and thereby reward-related learning [12, 13].

However, decision-making is not a unitary construct. Humans are equipped with a range of choice strategies, varying in accuracy and demand [14, 15]. Therefore, efficient decision-making requires us to allocate cognitive resources between these strategies [16, 17]. Even though such arbitration is critical for efficiently navigating the world [18, 19], it is unknown whether and how alcohol and substance abuse also impair this form of higher-order decision-making.

In this paper, we address this question through the lens of reinforcement learning (RL). Recent advances in RL have provided formalizations for two systems that control choice: a fast, automatic system and a slow deliberative system. In the language of machine learning, these systems are mapped to “model-free” and “model-based” RL [20–22]. Model-free strategies learn through trial-and-error, which is computationally cheap but inflexible. Model-based strategies are computationally demanding but more accurate because they plan through an internal model of the environment towards goals [23–26].

The arbitration between these systems follows a cost-benefit tradeoff. A greater influence of model-based decision-making is observed for high reward stakes to increase reward rate, but only when it is more accurate than model-free control [27, 28]. People rely more on model-free control when cognitive resources are taxed [29] or when planning complexity increases [30]. Moreover, the ability to arbitrate between these systems varies between populations: it emerges throughout adolescence [31] and declines during aging [32]. In non-human studies, it is observed that substance use alters the balance between model-free and model-based control in reward-related learning [33, 34]. Moreover, some studies in humans have shown that substance use results in dysfunctional and in some cases risk insensitive decision-making [35–37].

Here, we use this framework to study impairments in decision-making in individuals with long-term and frequent alcohol use [38, 39]. These impairments may be driven by an increased reliance on less taxing model-free control [7] or by reduced risk sensitivity (i.e., the trade-off between the value preference and the risk preference) [40]. However, others have reported that alcohol [41] and substance use [42] may be associated with reduced model-free decision-making, whereas some have reported comparable use of model-free and model-based control between Alcohol Users and Alcohol Non-Users [43]. However, it remains unknown whether alcohol use affects the arbitration between systems. That is, whether it is associated with a reduced ability to shift between strategies based contextual factors such as reward magnitude.

To examine these questions, we used a modified version of the “two-step task” [20, 24], a sequential decision-making paradigm that dissociates between model-based and model-free contributions to a choice. This task included a reward magnitude manipulation to test participants’ ability to increase model-based control when there is a heightened opportunity for reward [23].

We hypothesized that, compared to Alcohol Non-Users, (1) Alcohol Users would show less model-based decision-making in overall task performance, and (2) Alcohol Users would be less sensitive to reward amplification, showing less model-based decision-making in the high-stakes condition compared to the low-stakes condition. In addition, we also explored whether the hypothesized difference in arbitration is associated with different levels of risk sensitivity in Alcohol Users.

## Methods

### Participants

Eighty-one individuals (58% female) participated in an online study that was conducted during the COVID-19 epidemic (July 2020 – August 2022). The study was advertised on social media platforms and participants throughout the continental United States were eligible to participate in this study. All participants provided informed consent. The study was approved by the Institutional Review Board of the Icahn School of Medicine at Mount Sinai.

All participants completed the ‘Coronavirus Health Impact Survey’ (CRISIS) questionnaire [44]. Data on alcohol use frequency from the CRISIS survey item 146 (“*For the 3 months DURING LOCKDOWN, how frequently did you use alcohol* ?) was used to stratify participants into different groups. Participants who reported no alcohol use and those who reported consuming alcohol less than once a month were grouped as Alcohol Non-Users (*n* = 34), and those who reported consuming alcohol multiple times a month to more than once a day were grouped as Alcohol Users (*n* = 47). The Alcohol Use Disorders Identification Test (AUDIT) [45, 46], Patient Health Questionnaire PHQ-9) [47] and Generalized Anxiety Disorder Questionnaire (GAD-7) [48] were also used to assess the severity of alcohol use, depressive and anxiety symptoms, respectively.

### Modified Two-Step Task

The task used in this study is a modified version of a previously developed two-step learning task [24, 49], aimed to test whether choice behavior shows increased model-based control in the face of increased reward magnitude [27]. A detailed description of the task has been published previously [27].

**Fig. 1:**
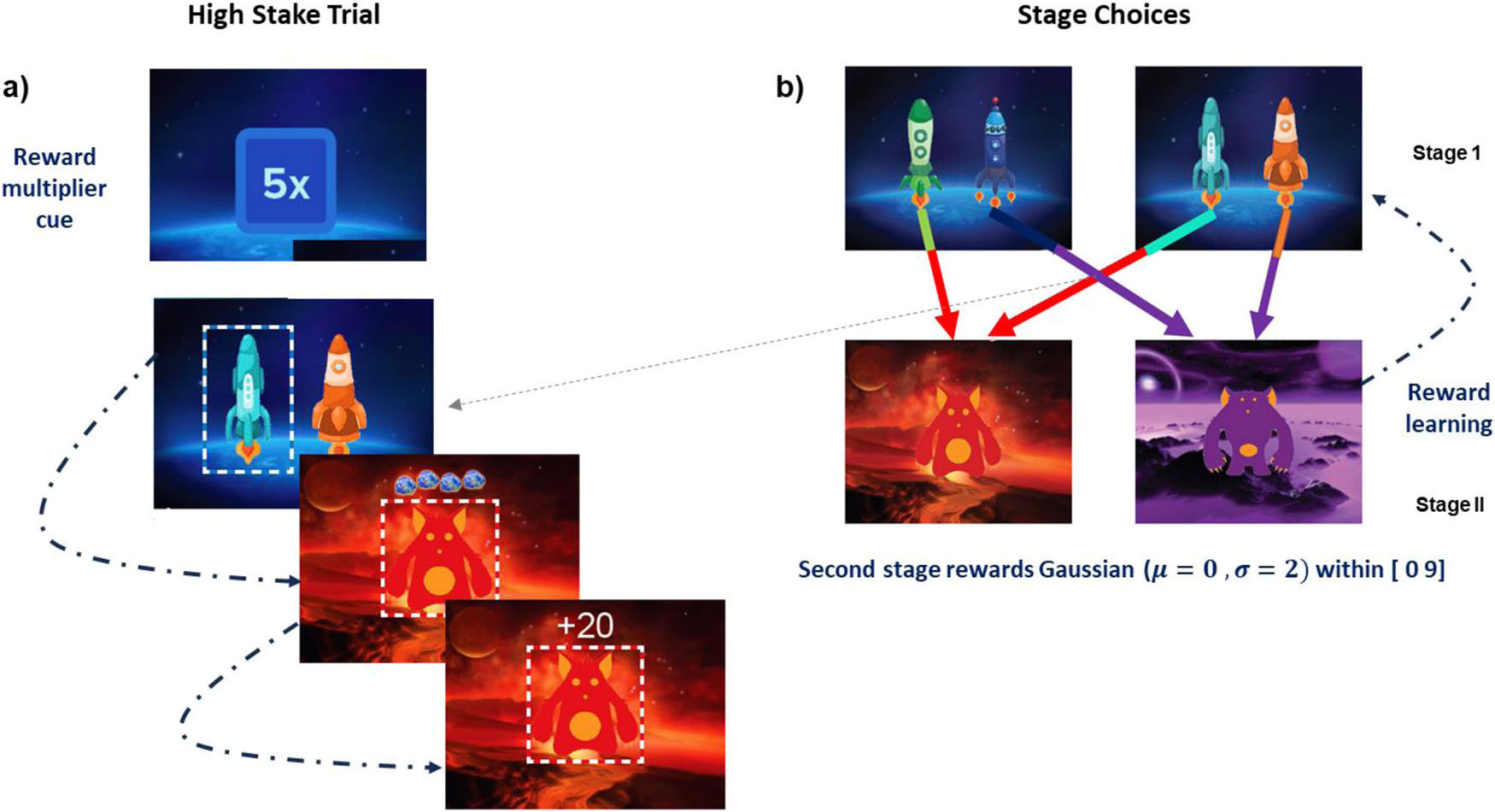
Modified 2-step learning task. **a** Cue indicating the nature of the trial by highlighting the stake multiplier 5x, indicating a highstakes. trial **b** Modified 2-step task: Participants choose the first-stage option between two spaceships, followed by a probabilistic transition to the second stage on either the red or purple planet [56].

Each trial starts randomly in one of two first-stage states, each with a choice between two spaceships that lead to a red or purple planet in the second stage (Fig. 1). These planets provide the opportunity to earn rewards. These independently drift over time for each planet. The task dissociates model-free from model-based decision making because each first-stage state offers an identical choice between transitioning to the red and the purple planet. A model-based decision-maker uses this equivalence to transfer experiences between states, while a model-free decision-maker relies on action-reward contingencies without considering the task’s transition structure [23, 27, 49].

Our version of this task included a reward magnitude manipulation [23]. Each trial started with a randomly picked cue that indicated whether it involved *low* or a *high* stakes. In high-stakes trials, the rewards earned in the second-stage state were multiplied by 5, whereas in low-stakes trials they were unaltered (Fig. 1). For each trial, there was a 50% probability it would involve low stakes, and 50% probability it would involve high stakes. This ensured there was no bias in the number of high- and low-stakes trials between the two groups of participants.

Each subject completed 200 trials. The task was programmed in jsPsych [50] and was hosted on Pavlovia platform (https://pavlovia.org). In addition to receiving compensation for participating in the study, participants were told that they could earn up to $5 based on their performance. However, in the end, all participants were paid $5 for completing this task.

### Computational modeling

#### Dual-system RL model

We used a dual-system RL model [24, 51, 52] to describe behavior in terms of both model-based and model-free control. The model-free system learns state-action values for all first and second-stage states through a simple temporal difference-learning algorithm [21], whereas the model-based system uses the transition structure of the task to plan towards goals. The relative tradeoff between these systems is modeled as a mixture between action values computed by these two systems.

The model consists of a function Q(s,a) that maps each state-action pair to estimates of future rewards. The task consists of four available states across two stages, two available actions at the first-stage states (a_A_ and a_B_) and one action at the second-stage states (a_C_).

The model-free system uses the SARSA(λ) temporal difference learning algorithm [53] to calculate values of each state-action pair. This algorithm updates the value of the state-action pair (s,a) at each stage ‘i’ and trial ‘t’, according to:

$${Q}_{{MF}}\left({s}_{i,t}{a}_{i,t}\right)={Q}_{{MF}}\left({s}_{i,t}{a}_{i,t}\right)+\alpha {\delta }_{i,t}{\varepsilon }_{i,t}(s,a)$$

where,

$${\delta }_{i,t}={r}_{i,t}+{Q}_{{MF}}\left({s}_{i+1,t},{a}_{i+1,t}\right)-{Q}_{{MF}}\left({s}_{i,t},{a}_{i,t}\right)$$

is the reward prediction error (RPE), α is the learning rate (determining the degree to which new information is incorporated), and ε_(i,t)_ (s, a) is the eligibility trace. The eligibility trace of each action is set to zero at the start of each trial and updated according to

$${\varepsilon }_{i,t}\left({s}_{i,t},{a}_{i,t}\right)={\varepsilon }_{i-1,t}\left({s}_{i,t},{a}_{i,t}\right)+1$$

before the Q-value update. After each update, the eligibilities of all state-action pairs are then decayed by λ. It is important to note that λ is a free parameter, but that ε is not. The former dictates the degree to which eligibilities are decayed, the latter simply counts which actions have been chosen.

Since there is no reward in the first state, the RPE (δ) for the first stage is only driven by the value of the second-stage action Q_MF_ (s_2,t_, a_2,t_):

$${\delta }_{1,t}={Q}_{{MF}}\left({s}_{2,t},{a}_{2,t}\right)-{Q}_{{MF}}\left({s}_{1,t},{a}_{1,t}\right)$$

Only the first-stage action is updated by this prediction error, and its eligibility for future updates on the current trial is only decayed by λ after this update.

Since there is no further stage beyond the second, the second-stage prediction error is only driven by the second-stage reward r_2,t_:

$${\delta }_{2,t}={r}_{2,t}-{Q}_{{MF}}\left({s}_{2,t},{a}_{2,t}\right)$$

Both the first- and second-stage values are updated at the second stage using this RPE. As mentioned above, the first-stage value is updated with the second-stage RPE down-weighted by the eligibility trace decay, λ. This means that, when λ = 0, only the value of the current second-stage action value is updated. When λ = 1, the values of the chosen first-stage and second-stage action values are updated by the same amount.

The model-based agent computes first-stage action values by combining a transition function, which maps the first-stage state-action pairs to a probability distribution over the subsequent states, with the second-stage (model-free) values. For each action a_j_ in first-stage state s_1,I_,these model-based values are defined in terms of the expected values of each first-stage action using the transition structure *P*:

$${Q}_{{MB}}\left({s}_{1,i},{a}_{j}\right)=P\left({s}_{2,1}|{s}_{1,i},{a}_{j}\right){Q}_{{MF}}\left({s}_{2,1},{a}_{c}\right)+P\left({s}_{2,2}|{s}_{1,i},{a}_{j}\right){Q}_{{MF}}\left({s}_{2,2},{a}_{c}\right)$$

At the second stage, the model-based and model-free coincide, and so ${Q}_{{MB}}={Q}_{{MF}}$.

To connect the values to choices, the Q-values of both systems are mixed according to a weighting parameter ω:

$${Q}_{{net}}({s}_{1,i},{a}_{j})={\omega }{Q}_{{MB}}({s}_{1,i},{a}_{j})+(1-{\omega }){Q}_{{MF}}({s}_{1,i},{a}_{j})$$

Thus, higher values of this parameter (closer to 1) reflect increased model-based control, whereas lower values (closer to 0) suggest stronger model-free control. To accommodate our stake manipulation, we defined two different weights for the different trial types. Specifically, we set ω = ω_low_ for low-stake trials and ω = ω_high_ for high-stake trials.

We used the soft-max rule to translate these Q-values to actions. This rule computes the probability for an action reflecting the mixture of action values weighted by an inverse temperature parameter. At both states, the probability of choosing action a on trial t is computed as:

$$p({a}_{i,t} = a|{s}_{i,t}) = \frac{\exp [\beta \left(\right.{Q}_{{net}}({s}_{i,t},a) + \pi \cdot {rep}(a) + \rho \cdot {resp}(a)]}{{\sum }_{{\rm{a}}^{\prime}} \exp [{\beta} \left(\right.{Q}_{net}({s}_{{i},{t}},{a^{\prime \prime}} ) + \pi \cdot {rep}({a^{\prime}} ) + {\rho} \cdot {resp} ({a^{\prime}})]}$$

where the inverse temperature *β* determines the randomness of the choice (with values close to zero reflecting fully random choice, and large positive values reflecting exploitation). The indicator variables *rep(a)* and *resp(a)* are set to 1 for actions or key presses that the participants chose on the previous trial and are zero otherwise. Multiplied with the ‘stickiness’ parameter π and ‘response stickiness’ parameter *ρ*, respectively, these capture the degree to which people show choice perseveration or switching at the first stage state.

We used a maximum *a posteriori* estimation with empirical priors based on prior work [32], implemented using the mfit toolbox [54] (https://github.com/sjgershm/mfit) to fit the free parameters in the computational models to observed data. For all parameters bounded between 0 and 1 ($\alpha ,{{\omega }}_{{low}},{{\omega }}_{{high}},\lambda ,\rho$) we used a Beta (2,2) prior. For the inverse temperature *β*, we used a Gamma (3, 0.2) prior and for the stickiness parameters $(\pi ,\rho )$, we used a ${\mathscr{N}}(\mathrm{0,1})$ prior. To avoid local optima in estimation, the optimization was run 100 times for each participant with random initializations for each parameter. The final estimations for all parameters were extracted from the run with the maximal posterior probability. (Supplementary table S1**)** reports the estimated parameters.

We also fit an “exhaustive” model that varied all parameters between the high- and low-stake trials. We used the same maximum a posteriori estimation with the same empirical priors as described above to obtain estimates for these 12 parameters.

#### Utility function

The utility function *U* [55] is a modified version of the utility formulation [56, 57] that estimates the risk sensitivity with varying reward magnitude and the trade-off between value preference and risk preference for a given state ‘s’ and action ‘a’. It is computed as:

$${U}_{t}(s,a) = {R}_{t}(s,a)-\mu .{sign}({R}_{t}(s,a)) \cdot {\sqrt {h}}_{t}(s,a)$$

Here, the risk sensitivity parameter *μ* reflects risk preference, the term *sign*(*R*_*t*_(*s*,*a*)) reduces the magnitude of estimation function for positive values of *R* and increases it for negative values of *R*, and the return variance or risk function (√h_t_). Thus, this function naturally incorporates the notion of increased risk-seeking behavior for gains and increased risk-aversive behavior for losses.

This function estimates five parameters μ (risk sensitivity), β (explore-exploit tradeoff), η (learning rate), Γ (discount factor for long-term rewards), and δ_limit_ (maximum error magnitude associated with reward). Higher μ implies risk aversion and lower value implies risk seeking, higher β favors exploitation, η is the learning rate, Γ weighs long-term over immediate rewards, and δ_limit_ is the maximum error magnitude.

For risk-value estimation, the model only considers first stage states since the second stage action does not impact the risk trade-off. We fit parameters separately for low- and high-stake trials, using the genetic algorithm [58] to identify the best fit considering response changes (mutations), learning adjustments (crossovers) and choices. The action selection is based on soft-max probabilities, where the expected return is updated using a learning rate $(\eta )$ and a temporal difference error $(\delta )$. The genetic algorithm evolves mutations and the crossovers over the initial population (size = 1000, generations = 100) to optimally fit parameter values. The free parameters are estimated by maximizing the fitness function iterated over 200 trials.

Of the five free parameters, the risk sensitivity parameter $(\mu )$ was of most interest for statistical analysis. Therefore, the **Supplementary Section** provides additional details of the utility function. Here, we have used two different models (1) to estimate the degree of model-based vs model-free control, and (2) to estimate risk sensitivity. Given the relatively small sample size of Alcohol Users (*n* = 47) and Alcohol Non-users (*n* = 34), to ensure the reliability of results, we did not split the data further into test and training sets to cross-validate the model accuracy.

#### Statistical approach

Parameter fits were analyzed using a 2 × 2 analysis of variance (ANOVA) using stakes (High vs. Low) and group (Alcohol users vs. Alcohol Non-Users) as between-subject factors. Since the parameter fits of both models were not normally distributed, we used the Mann-Whitney U test for hypothesis testing. Furthermore, we used Spearman Rank correlation to assess the associations between model-based and model-free weighting $(\omega )$ and risk sensitivity ($\mu$) parameters (calculated for high-stakes trials, low-stakes trials, and the difference between high- and low-stakes trials) with each other and with validated measures of alcohol use severity (i.e., AUDIT scores) and depression symptom severity (i.e., PHQ-9 scores). Finally, we performed a correlation analysis to investigate the relationship between the weighting parameter (ω) and risk sensitivity (μ) obtained from two models. The analyses were conducted first for the entire sample and then separately for Alcohol Users and Alcohol Non-Users. Bonferroni corrections were applied to adjust for multiple comparisons. To assess the robustness of these correlations, as both parameters are derived from the same dataset, we employed a cross-validation approach by splitting each subject’s dataset into two halves. We conducted two types of correlation analyses: one by splitting the data into the first 100 trials and the second 100 trials for preserving temporality, and the other by separating all odd-numbered trials from even-numbered trials, correlating ω and μ derived from these distinct datasets. Results from these cross-validations, along with the sensitivity analysis are presented in the Supplemental Section.

## Results

### Sample characteristics

As shown in Table 1, groups did not differ significantly in gender (*p* =0.282), ethnicity (*p* =.529), and education (*p* = 0.943). However, they significantly differed in age (*p* < 0.001), such that a greater proportion of participants (Alcohol Non-Users: 73%, Alcohol Users: 61%) were between the ages 18 and 40. As expected, AUDIT scores were significantly higher in Alcohol Users, compared to Alcohol Non-Users (*p* < 0.001), showing more severe alcohol use in Alcohol Users (Mean and standard deviation are reported in Table 1). Age did not correlate significantly with computational variables, and therefore, was not used as a covariate. We report the results with age as a covariate in the **Supplementary Section**.

**Table 1:** Demographics of Alcohol Non-Users and Alcohol Users

Demographic Details	Alcohol Non- Users	Alcohol Users	t-test statistics
	*n* = 34	n =47	
**Gender**			
Male	11 (0.32)	6 (0.26)	t(77) = 1.083, *p* = 0.282
Female	22 (0.65)	17 (0.74)	
Other	1 (0.03)	4 (0.09)	
**Age**			
<18 Years	9 (0.26)	2 (0.04)	t(75) = −4.395, *p* < 0.001
18–19 Years	4 (0.12)	4 (0.08)	
20–21 Years	4 (0.12)	2 (0.04)	
22–23 Years	4 (0.12)	12 (0.26)	
24–5 Years	1 (0.03)	2 (0.04)	
25–40 Years	12 (0.35)	9 (0.19)	
>40 Years	0 (0)	12 (0.26)	
**Hispanic**	6 (0.18)	10 (0.21)	t(75) = −0.596, *p* = 0.553
**Race**			
White	19 (0.56)	26 (0.56)	t(73)= −0.638, *p* = 0.529
Black	1 (0.03)	7 (0.15)	
Other	8 (0.23)	14 (0.3)	
**Education**			
High School /Diploma/GED	10 (0.3)	13 (0.28)	t(75) = −0.072, *p* = 0.943
College degree/ associate degree	16 (0.47)	19 (0.4)	
Post Graduate degree	8 (0.24)	11 (0.23)	
**Mental Health Diagnosis**	12 (0.35)	16 (0.34)	t(61) = −0.930, *p* = 0.356
AUDIT*	19 (0.89 ± 0.21)	40 (8.5 ± 12.32)	*p* < 0.001

^*^Continuous variables are represented by mean and standard deviation, while categorical variables are represented by percentages.

### Dual system RL model outcomes

Here, we report the statistical tests on variables that relate most closely to our hypotheses, namely the average reward in the task, and the degree of model-based control $(\omega )$. The analyses on the other RL parameters are presented in the **Supplementary Section**.

A between-group Mann-Whitney U test showed no significant group differences in mean reward rate (*Z* = 1.024, *p* = 0.306) or the average number of points collected per trial. This suggests that, on average, groups performed equally well.

**Fig. 2:**
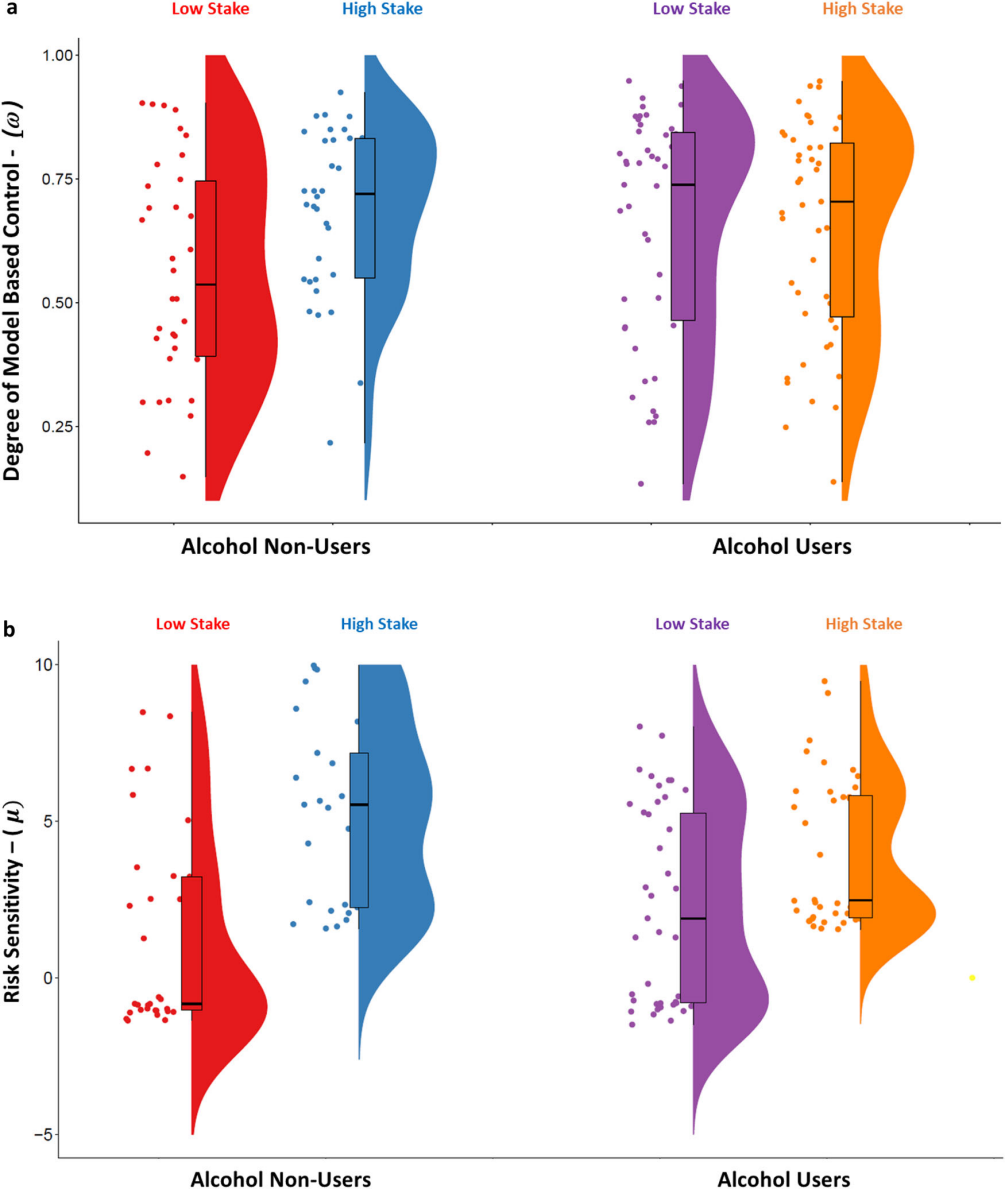
Group differences in reward-related modulation in weighting parameter and risk sensitivity modulation. **a** Weighting parameter reflecting model-based decision control. **b** Risk sensitivity measure determining the tradeoff between risk avoidance and risk seeking.

However, a 2 (Stakes: Low, High) × 2 (Groups: Alcohol Non-Users, Alcohol Users) ANOVA showed a significant main effect of Stakes (*F*_79,1_ = 5.469, *p* = 0.022, partial *η*^*2*^ = 0.065) and a Stakes × Groups interaction (*F*_79,1_ = 6.463, *p* = 0.013, partial *η*^*2*^ = 0.076) on model-based control. The main effect of Group (*F*_79,1_ = 0.504, *p* = 0.480, partial *η*^*2*^ = 0.006) was not statistically significant. Follow-up within-subject Wilcoxon tests revealed that the difference between the High and Low model-based weighting parameters was statistically significant in Alcohol Non-Users (*Z* = −2.402, *p* = 0.016), but not in Alcohol Users (*Z* = −0.508, *p* = 0.611). Mann-Whitney U tests revealed no significant between-group difference in mixing weight for Low (*Z* = −1.876, *p* = 0.061) nor High (*Z* = −0.612, *p* = 0.540) stakes (Fig. 2a). This result suggests that, even though performance on the task was comparable, Alcohol Users were less likely to adopt different strategies based on the reward incentive.

### Utility Function

Next, we turned our attention to our measure of risk sensitivity from the utility function. A 2 x 2 ANOVA of showed a significant main effect of Stakes (*F*_76,1_ = 36.08, *p* < 0.001, partial *η*^*2*^ = .322) and a Stakes × Groups interaction (*F*_76,1_ = 4.524, *p* = 0.037, partial *η*^*2*^ = 0.056). There was no significant main effect of Group (*F*_76,1_ = .992, *p* = 0.322, partial *η*^*2*^ = 0.013). Post-hoc analyses using independent *t*-tests found that risk sensitivity did not differ between groups on low stakes trials (*t* = −1.461, *p* = 0.152), but that on high stakes trials risk sensitivity was significantly higher in Alcohol Non-Users compared to Alcohol Users (*t* = 2.280, *p* = 0.030). Follow-up within-subject Wilcoxon tests revealed that the risk sensitivity difference between the High and Low stake trail was statistically significant both in Alcohol Non-users (*Z* = −3.702, *p* < 0.001), and in Alcohol Users (*Z* = −2.737, *p* = 0.006). Mann-Whitney U tests revealed a significant between-group difference in risk sensitivity for High (*Z* = −2.016, *p* = 0.044) and a significant trend for Low (*Z* = 1.950, *p* = .051) stakes. This result suggests that both Alcohol Users and Alcohol Non-users were sensitive to risk and reward magnitude (Fig. 2b).

### Correlation analyses

**Fig. 3:**
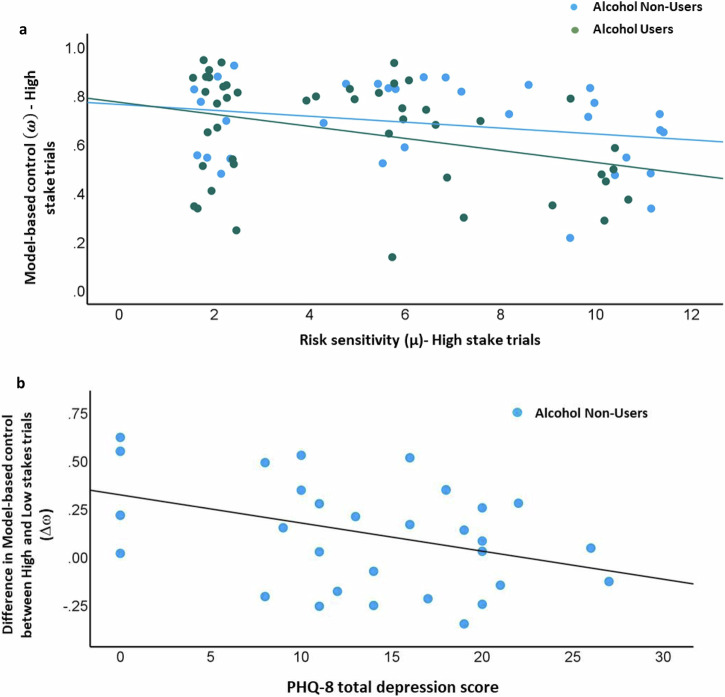
Correlations between model parameters and clinical variables. **a** Correlation between model-based vs -free weighting parameter and Risk Sensitivity during high stakes trials in the total sample, as a function of Alcohol User and Non User group membership. **b** Correlation between model-based vs -free weighting parameter and severity of depressive symptoms in the Non User sample.

Finally, we explored the relation between the parameters from the two computational models. Correlation analyses revealed that for High stakes trials in Alcohol Users, the weighting parameter (ω) was negatively correlated with risk sensitivity (*μ*) (*r* = −0.349, *p* = 0.018), such that in Alcohol Users, less model-free control was associated with greater risk aversive behavior. However, this relationship was not statistically significant in Alcohol Non-Users (*r* = −0.229, *p* = 0.207) (Fig. 3a). Further, Fisher’s Z transformation indicated that these correlations were not significantly different between Alcohol Users and Alcohol Non-users (z = −0.55, *p* = 0.5823). Moreover, in Alcohol Non-Users, the greater difference between mixing weight for High relative to Low stakes (Δω) was associated with lower depressive symptoms (assessed via the PHQ-8; *r* = −0.360, *p* = 0.040), such that Alcohol Non-Users with lower depressive symptoms showed greater increase model-based decision making in high- relative to low-stakes trials (Fig. 3b). (Supplemental table S2 has additional analysis)

## Discussion

This study used a modified two-step task to examine the arbitration between model-based and model-free RL systems in Alcohol Users compared to Alcohol Non-Users. We found that Alcohol Non-Users and Alcohol Users did not differ in the overall reliance on model-based control during the task. However, unlike Alcohol Non-Users, Alcohol Users did not increase model-based control based on reward incentives. Additionally, even though both groups exhibited increased risk aversion in high- compared to low-stakes trials, this effect was pronounced for Alcohol Users. Lastly, in Alcohol Users greater model-based control was associated with less risk aversiveness in high reward stakes, and in Alcohol Non-Users greater increase in model-based control in high relative to low reward conditions was associated with lower depressive symptoms.

We have found evidence that alcohol use impairs the ability to arbitrate between decision making strategies. Alcohol Non-Users increased their use of model-based control on high-stakes trials, consistent with prior reports using healthy young adults [23, 31, 32]. However, Alcohol Users did not show such flexibility in arbitration. These findings suggest that people who use alcohol regularly struggle to shift between decision-making strategies based on contextual factors such as reward-related anticipatory cues and therefore make less optimal choices. Of course, arbitration between strategies is not only based on reward incentives. Inflexible arbitration between model-based and model-free control generalizes to other external influences. Indeed, a recent study showed that while Alcohol Non-Users employed less model-based control in high- compared to low-stress conditions, Alcohol Users did not show such a reduction in model-based control across stress levels [59]. Future work, varying other contextual variables, will broaden our understanding of how alcohol use affects controlled arbitration.

Importantly, some studies have previously shown greater reliance on model-free compared to model-based control in substance [42] and alcohol [41] users. We did not find such group differences. This can potentially be explained by differences in the task. In our modified two-step task, model-based control is more accurate than model-free control, which is a feature that is absent in prior versions of the task [27]. Moreover, model-based planning is more demanding in the older version, requiring reasoning over stochastic transitions (compared to the deterministic transitions in our version). Nevertheless, a recent study using young adults provided convergent evidence for our findings, in the form of a lack of an association between alcohol use and model-based control [43].

The reward-magnitude manipulation in our task also changed participants’ explore-exploit tradeoff (results are presented in Supplementary Section). On high-stakes trials, participants reduced exploration and increased exploitation, picking the high-value action in the first stage of our task, instead of choosing the alternative. This pattern, which we have also previously reported in healthy individuals [23], did not differ between the Alcohol Users and Alcohol Non-Users. This finding assuages the potential concern that Alcohol Users simply become less sensitive to rewards and were therefore less inclined to use more model-based control. Instead, these results reinforce the idea that Alcohol Users are less flexible in arbitrating between RL strategies. However, a previous study found reduced exploratory behavior in individuals with alcohol use disorder [60]. Unfortunately, differences between this task and ours limit our ability to make direct comparisons.

Our results also showed that Alcohol Users showed stronger transfer of reward learning from the second-stage to the first-stage of the task. In other words, the RPE in the second stage of the task had a comparatively greater influence on reward expectations in the first-stage state of the following trial for users compared to Alcohol Non-Users. This means that the model-free systems of the Alcohol Users reflect outcomes of the second stage from the previous trials more so than it does in Alcohol Non-Users. This finding is consistent with previous research indicating that individuals with a history of substance and alcohol use encounter difficulties in transferring their learning effectively [61–64]. Specifically, they tend to make random choices, relying on past experiences to judge the effectiveness of new observations and behaviors [35, 65]. The overall implication is that substance and Alcohol Users apply acquired knowledge differently in novel situations, possibly due to challenges in making adaptive decisions.

We also documented that risk sensitivity was higher for the high- relative to low-stakes conditions for both groups, which is consistent with prior literature [66]. Interestingly, however, although Alcohol Users showed greater risk aversiveness in high- compared to low-stakes trials, their risk aversiveness for high stakes was still lower than that of Alcohol Non-Users, and the increase in risk aversiveness from low- to high-stakes trials was lower than that in Alcohol Non-Users. These results are consistent with prior studies showing that Alcohol Users show more risky decision making in high-reward magnitude contexts compared to Alcohol Non-users [35]. Moreover, although model-based control is typically associated with risk aversion and loss aversion [67], our correlational results revealed that in Alcohol Users, greater model-based control was associated with lower risk aversiveness during high reward stakes, although the result did not survive validation analyses. This behavior may be influenced more by emotional instead of cognitive assessment of risk [68–70]. This highlights the notion of maladaptive decision-making strategies/models employed by Alcohol Users [59], greater use of which is associated with riskier decision-making.

The results of this study should be viewed considering some potential limitations. First, the characterization of alcohol use which led to group stratification was determined based on ordinal self-reported data from the CRISIS questionnaire, a measure designed to assess changes in substance use and psychiatric symptoms during the COVID-19 pandemic-related lockdowns and our survey data was all collected during the pandemic time window. Given our stratification measure and time window of data collection, the alcohol use group included in our study may reflect a stress-induced heavy-drinking population more so than a heavy-drinking population in the absence of external stressors. Thus, the generalizability of our sample to other populations may be another limitation. However, it is important to note that the group characterization was supported by significantly higher AUDIT scores in Alcohol Users compared to Alcohol Non-Users, validating the group stratification based on alcohol use severity. However, comparison of results with those from prior studies is limited since the clinical characteristics beyond AUDIT scores were not assessed in this study. Second, even though our results show that alcohol use diminishes the ability to shift between RL strategies, it remains unclear why this is the case. One possibility is that Alcohol Users are impaired in estimating the relative values of both habitual and goal-directed strategies, leading to reduced uncertainty about the appropriate strategy in dynamically changing contexts. Another possibility is that Alcohol Users attach a higher cost to switching between strategies, which would suggest that their lack of arbitration reflects a motivational deficit. Even though our data are unable to distinguish between these explanations, they invite a novel research program aimed at doing so. The third limitation of this survey-based study is the lack of assessment of fluid intelligence and working memory. This could have influenced the task performance and therefore limits the generalizability of these results. Nevertheless, these results provide an evidence-based foundation for future studies that should assess alcohol use and other determinants of decision making in more detail to examine the impact of frequency and recency of alcohol use on reward-related decision making. Fourth, the lack of assessment of other specific mental health disorders may have confounded these results. The CRISIS questionnaire includes data on whether participants had any history of mental health diagnoses (binary: yes or no), and the proportion of those with mental health prior diagnoses was comparable between the two groups. Lastly, this study could have benefited from a larger sample size with varying alcohol use severity such that computational RL models can be effectively employed to understand the relationship of reward-related decision making and alcohol use severity.

## Conclusions

We have shown that frequent alcohol use is associated with less flexible arbitration between goal-directed (i.e., model-based) and habit-based (i.e., model-free) control in situations where reward magnitude increases, and with lower risk aversiveness in high reward magnitude contexts. Although we expected greater goal-directed control to be associated with greater risk aversiveness, this relationship was inverted in Alcohol Users suggesting that the decision models developed in Alcohol Users may not only be rigid to varying reward values, but also associated with risky decision making. Results from this behavioral study paves the way for neuroimaging studies to further examine neurobiological underpinnings of maladaptive reward-related decision-making strategies in Alcohol Users and to examine these effects in other substance and/or behavioral addictions.

## Supplementary information

Supplementary Material

## Data Availability

The datasets and scripts generated and analyzed during the current study are available in the maplab@mssm.edu at https://osf.io/e5tvs/.
