# Supplementary material for: Impaired arbitration between reward-related decision-making strategies in Alcohol Users compared to Alcohol Non-Users: a computational modeling study
Source: NPP Digit Psychiatry Neurosci. 2025 Jan 3;3:1. doi: 10.1038/s44277-024-00023-8 (PMC11698690; doi:10.1038/s44277-024-00023-8)
Supplement: Supplementary file 1 — Supplementary Material [file 44277_2024_23_MOESM1_ESM.docx]

**Supplemental Online Information**

**Impaired arbitration between reward-related decision-making strategies in alcohol users compared to non-users: A computational modeling study.**

Srinivasan Anantha Ramakrishnan, PhD^1,2*^; Riaz B. Shaik, PhD^2*^; Tamizharasan Kanagamani MS^3^; Gopi Neppala, BA^2^; Jeffrey Chen, BA^4^; Vincenzo Fiore, PhD^2^; Christopher J. Hammond, MD, PhD^5^; Wouter Kool, PhD^6^; Shankar Srinivasan, PhD^1^; Iliyan Ivanov, MD^2^; Srinivasa Chakravarthy PhD^3^; Muhammad A. Parvaz, PhD^2,7,8^

^1^ Department of Health Informatics, Rutgers University, New Brunswick, NJ

^2^ Department of Psychiatry, Icahn School of Medicine at Mount Sinai, New York, NY

^3^Department of Biotechnology, Indian Institute of Technology, Madras, Chennai, India

^4^University of Pittsburgh School of Medicine, Pittsburgh, PA

^5^Department of Psychiatry & Behavioral Sciences, Division of Child & Adolescent Psychiatry, Johns Hopkins University School of Medicine, Baltimore, MD

^6^Department of Psychological & Brain Sciences, Washington University in St. Louis, St. Louis, MO

^7^Department of Neuroscience, Icahn School of Medicine at Mount Sinai, New York, NY

^8^Department of Artificial Intelligence and Human Health, Icahn School of Medicine at Mount Sinai, New York, NY

*** denotes equal contribution as co-first authors**

**Corresponding Author:**

Muhammad A. Parvaz, PhD

Assistant Professor

Departments of Psychiatry, Neuroscience, and Artificial Intelligence and Human Health

Icahn School of Medicine at Mount Sinai

One Gustave L. Levy Place, Box 1230

New York, NY 10029

Email : [Muhammad.parvaz@mssm.edu](mailto:Muhammad.parvaz@mssm.edu)

Phone : 212-241-3638

Fax : 212-241-1504

**Disclosures**: The authors report no conflict of interest concerning the materials or methods used in this study or the findings specified in this paper.

**Funding**: This research was supported by the National Institute on Drug Abuse (K01DA043615, R01DA058039, and R61DA056779, to MAP).

1. **Utility Function**

The Utility function (U), modified from the original formulation used in [1, 2], estimates the trade-off between value preference and risk preference [3] for a given state (s) and action (a) as follows:

| $U_{t}\left( s,a \right)=R_{t}\left( s,a \right)-\mu.sign\left( R_{t}\left( s,a \right) \right).\surd h_{t}\left( s,a \right)$ |  |
| --- | --- |

Risk preference consists of the risk sensitivity parameter *μ*, the term *sign*(*R_t_*(*s*,*a*)) reduces the magnitude of estimation function for positive values of *R* and increases it for negative values of *R*, and the return variance or risk function (√h_t_). Thus, this function naturally incorporates the notion of increased risk-seeking behavior for gains and increased risk-aversive behavior for losses.

This function analyzes five parameters: $\mu, \theta, \gamma, \eta, and \deltaₗᵢₘᵢₜ.$ The parameter μ represents risk sensitivity, where higher values lead to risk-averse actions and lower values lead to risk-seeking actions. The parameter $\beta$ controls the explore-exploit tradeoff with higher values indicating more exploitation and lower values indicating higher exploration. The parameter $\eta$ is the learning rate, and. The discount factor Γ accounts for the relative importance of long-term rewards compared to immediate rewards. Finally, δₗᵢₘᵢₜ represents the maximum magnitude of the error, and has previously shown to be associated with dopamine availability [4].

For risk-value estimation, model only considers first stage states since the second stage action does not impact the risk prediction outcomes. Parameters are fit separately for low- and high-stake trials, and the genetic algorithm [5] is used to identify the best fit considering response changes (equivalent to mutations), learning adjustments (equivalent to crossovers) and the choices.

The action selection is based on the probabilities estimated by the soft-max, whereas the expected return (*Rₜ*) is updated using a learning rate $(\eta)$ and a temporal difference error $(\delta)$.

$$R_{t}\left( s,a \right)=R_{t}\left( s,a \right)+\eta\delta$$

The temporal difference error $(\delta)$ is calculated by the current reward (r) added to the difference between the discounted next expected return $(\gamma Rₜ₊₁(s', a'))$ and the current expected return $(Rₜ(s, a)).$

$$\delta=r+\gamma R_{t+1}(s^{'},a^{'})-R_{t}(s,a)$$

Subsequently, the return variance ($hₜ)$is updated, where $\etaₕ$represents the learning rate and $\zeta$is the squared temporal difference error $(\delta^{2})$subtracted from $hₜ.$

$$h_{t}\left( s,a \right)=h_{t}\left( s,a \right)+\eta_{h} \zeta$$

$$\zeta=\delta^{2}-h_{t}(s,a)$$

The probability $P\left( a | s \right)$of selecting action a given state s is determined, using soft-max policy, where $\beta$is the inverse temperature parameter.

$$P(a|s)=\frac{e^{{\beta U}_{t}(s,a)}}{\sum_{i} e^{\beta U_{t}(s,a_{i})}}$$

| $P(a\vert s)=\frac{e^{\beta U_{t}(s,a)}}{\sum_{i} e^{\beta U_{t}(s,a_{i})}}$ |
| --- |

The genetic algorithm evolves mutations and the crossovers over the initial population (Population size 1000, Number of generations 100) with n number of genes (free parameters for high and low stakes) to optimally fit parameter values. Initially, the random values are generated for the parent’s generation.

| $F=\frac{1}{N}\sum_{t=1\ldots N} \sum_{i=1..4} {\vert\mu}_{t,i}^{m}-\mu_{t,i}^{p}\vert\sum_{t=1\ldots N} \sum_{i=1..4} {\vert a\mu}_{t,i}^{m}-{\mu a}_{t,i}^{p}\vert$ |
| --- |

## The free parameters are estimated by minimizing the fitness function iterated over trials t (N=200). Here, $\boldsymbol{a}_{\boldsymbol{t}}^{\boldsymbol{m}}$ denotes the vector representation of actions selected by the model and $\boldsymbol{a}_{\boldsymbol{t}}^{\boldsymbol{p}}$ denotes the actions selected by the participants in a trial. While Utility function estimated five free parameters, we are only interested in the risk sensitivity parameter ($\boldsymbol{\mu}\mathbf{)}$ for statistical analysis

1. **Best fitting model parameters.**

|  | **Alcohol Non-Users** | **Alcohol User** |
| --- | --- | --- |
| Dual System RL Model | | |
| Reward rate corrected | 0.38 ± 0.05 | 0.44 ± 0.05 |
| Inverse temperature low | 0.4 ± 0.05 | 0.43 ± 0.03 |
| Inverse temperature high | 0.6 ± 0.05 | 0.54 ± 0.04 |
| Learning rate low | 0.62 ± 0.05 | 0.64 ± 0.04 |
| Learning rate high | 0.64 ± 0.05 | 0.64 ± 0.04 |
| Eligibility trace decay low | 0.5 ± 0.02 | 0.55 ± 0.02 |
| Eligibility trace decay high | 0.48 ± 0.02 | 0.55 ± 0.02 |
| Mixing Weight Low | 0.56 ± 0.04 | 0.66 ± 0.03 |
| Mixing weight high | 0.69 ± 0.03 | 0.65 ± 0.03 |
| Choice stickiness low | 0.16 ± 0.12 | 0.42 ± 0.1 |
| Choice stickiness high | 0.34 ± 0.09 | 0.53 ± 0.09 |
| Response stickiness low | -0.15 ± 0.13 | -0.3 ± 0.08 |
| Response stickiness high | -0.21 ± 0.13 | -0.31 ± 0.08 |
| Utility function – Genetic model | | |
| Risk sensitivity low | 1.27 ± 0.56 | 2.3 ± 0.45 |
| Risk sensitivity high | 6.54 ± 0.63 | 4.82 ± 0.45 |
| (Gen) Learning rate low | 0.46 ± 0.06 | 0.51 ± 0.05 |
| (Gen) Learning rate high | 0.38 ± 0.07 | 0.39 ± 0.05 |
| Discount Factor low | 0.52 ± 0.05 | 0.53 ± 0.04 |
| Discount Factor high | 0.51 ± 0.05 | 0.58 ± 0.05 |
| (Gen) Inverse Temperature low | 0.42 ± 0.05 | 0.5 ± 0.04 |
| (Gen) Inverse Temperature high | 0.53 ± 0.05 | 0.52 ± 0.04 |
| Temporal difference error limit low | 0.98 ± 0.32 | 1.27 ± 0.31 |
| Temporal difference error limit high | 1.09 ± 0.33 | 1.08 ± 0.29 |
| Temporal difference error limit high-low | 0.11 ± 0.43 | -0.19 ± 0.43 |

**Table 2:** Demographics of Alcohol Non-Userss and Alcohol Users

**Table S1: Best fitting model parameter**

1. **Dual System RL Model Outcomes**

***Inverse Temperature (β)***

The 2 × 2 ANOVA showed a statistically significant main effect of Stakes, (*F*_79,1_ = 23.13, *p* < .001, partial *η^2^* = .226) such that the inverse temperature (i.e., exploitation relative to exploration) was higher for High, compared to Low, Stakes condition across both groups. However, the Group main effect (*F*_79,1_ = 0.149, *p* = .701, partial *η^2^* = .002) and the Stakes × Groups interaction (*F*_79,1_ = 1.737, *p* = .191, partial *η^2^* = .022) were not statistically significant.

***Eligibility Trace (λ )***

The 2 × 2 ANOVA showed no significant main effect of Stakes (*F*_79,1_ = .555, *p* = .458, partial *η^2^* = .007) and no significant Stakes × Groups interaction (*F*_79,1_ = .763, *p* = .385, partial *η^2^* = .010). However, the Group main effect (*F*_79,1_ = 7.099, *p* = .009, partial *η^2^* = .082) was significant, such that the eligibility trace was higher in Alcohol Users compared to Alcohol Non-Users.

***Learning Rate (α)***

The 2 × 2 ANOVA showed no significant main effects of Stakes (*F*_79,1_ = .122, *p* = .727, partial *η^2^* = .002) and Group (*F*_79,1_ = .015, *p* = .901, partial *η^2^* = .000) and no significant the Stakes × Groups interaction (*F*_79,1_ = .354, *p* = .554, partial *η^2^* = .004).

***Choice Stickiness (π )***

The 2 × 2 ANOVA showed no significant main effects of Stakes (*F*_79,1_ = 3.416, *p* = .068, partial *η^2^* = .041) and Group (*F*_79,1_ = 3.628, *p* = .060, partial *η^2^* =.044), and no significant Stakes × Groups interaction (*F*_79,1_ = .0.151, *p* = .699, partial *η^2^* = .002).

***Response Stickiness (ρ )***

The 2 × 2 ANOVA showed no significant main effects of Stakes (*F*_79,1_ = .376, *p* = .542, partial *η^2^* = .005) and Group (*F*_79,1_ = .982, *p* = .325, partial *η^2^* = .012), and no significant Stakes × Groups interaction (*F*_79,1_=.190, *p* = .664, partial *η^2^* = .002).

1. **Dual System RL Model Outcomes -** *Model Outcomes with age as a covariate )*

In the statistical analysis, we compared model parameters with age as covariate to confirm additional sensitivity for extent of model based - model free arbitration $(\omega)$, exploration and exploitation trade-off $(\beta)$, the transfer of learning $\left( \lambda\right),$the learning rate $(\alpha)$, degree of repetitiveness of choices $\left( \pi\right)$and responses $(\rho)$, and the risk sensitivity $(\mu)$

***Model-based/model-free weighting parameter (ω )***

The 2 (Stakes: Low, High) × 2 (Groups: Alcohol Non-Users, alcohol users) ANOVA showed a significant main effect of Stakes (*F*_74,1_ = . 5.957, *p* = .017, partial *η^2^* = .075) . However, Stakes × Groups interaction (*F*_74,1_ = 3.347, *p* = .071, partial *η^2^* = .043) and Group main effect (*F*_74,1_ = 0.844, *p* = .361, partial *η^2^* = .011 ) were not statistically significant. Follow-up with independent t-test revealed that the difference between the High and Low weighting parameters were statistically not significant both in Alcohol Non-Users (*t(79) = -1.860, p = 0.067)*  and in Alcohol Users (*t(79) = 0.832, p = .408* ). Mann-Whitney U tests revealed no significant between-group difference in mixing weight for Low (*Z* = -1.876, *p* = .061) nor High (*Z* = -.612, *p* = .540) stakes

***Inverse Temperature (β)***

The 2 × 2 ANOVA showed a statistically significant main effect of Stakes, (*F*_74,1_ = 14.382, *p* < .001, partial *η^2^* = .163) such that the inverse temperature (i.e., exploitation relative to exploration) was higher for High, compared to Low, Stakes condition across both groups. However, the Group main effect (*F*_74,1_ = .245, *p* = .622, partial *η^2^* = .003) and the Stakes × Groups interaction(*F*_74,1_ = .245, *p* = .622, partial *η^2^* = .003) were not statistically significant.

***Eligibility Trace (λ )***

The 2 × 2 ANOVA showed no significant main effect of Stakes (*F*_74,1_ = .037, *p* = .848, partial *η^2^* = .000) and no significant Stakes × Groups interaction (*F*_74,1_ = .728, *p* = .396, partial *η^2^* = .010) . However, the Group main effect (*F*_74,1_ = 6.626, *p* = .012, partial *η^2^* = .082) was significant, such that the eligibility trace was higher in Alcohol Users compared to Alcohol Non-Userss.

***Learning Rate (α)***

The 2 × 2 ANOVA showed no significant main effects of Stakes (*F*_74,1_ = .120, *p* = .729, partial *η^2^* = .002) and Group (*F*_74,1_ = .275, *p* = .602, partial *η^2^* = .004) and no significant the Stakes × Groups interaction (*F*_74,1_ = .701, *p* = .405, partial *η^2^* = .009) .

***Choice Stickiness (π )***

The 2 × 2 ANOVA showed no significant main effects of Stakes (*F*_74,1_ = .184, *p* = .669, partial *η^2^* = .002) and Group (*F*_74,1_ = 2.688, *p* = .105, partial *η^2^* = .035) , and no significant Stakes × Groups interaction (*F*_74,1_ = .171, *p* = .681, partial *η^2^* = .002) .

***Response Stickiness (ρ )***

The 2 × 2 ANOVA showed no significant main effects of Stakes (*F*_74,1_ = .207, *p* = .651, partial *η^2^* = .003) and Group (*F*_74,1_ = .888, *p* = .349, partial *η^2^* = .012) , and no significant Stakes × Groups interaction (*F*_74,1_ = .893, *p* = .348, partial *η^2^* = .012) .

## Utility Function

***Risk Sensitivity from the risk – value estimation Function*** *(*$\boldsymbol{\mu}$*)*

The 2 x 2 ANOVA of risks sensitivity parameter estimated using the utility function, showed significant Stakes main effect (*F*_71,1_ = 15.589, *p* < 0.001, partial *η^2^* = .180) follow up with Mann-Whiteny parametric test showed risk sensitivity was significant in high stakes (Z = -2.016, p = .044) and not (trending) in low stakes (Z = -1.950, p = .051). However, there was no significant Stakes × Groups interaction (*F*_71,1_ = 1.715, *p = .195*, partial *η^2^* =.024) and Groups main effect (*F*_71,1_ = .409 , *p = .524*, partial *η^2^* =.006).

1. **Additional Correlation analysis**

| **Alcohol Non-User and Alcohol Users** | | Z (Coefficient) | P |  |
| --- | --- | --- | --- | --- |
|  |  |  |  |  |
| Risk sensitivity (μ)  Low Stake Trial ( n = 78 ) | Weighting parameter (w) -Low Stake trial | 0.064 | 0.580 |  |
|  | Weighting parameter (w) High Stake trial | 0.136 | 0.235 |  |
|  | Weighting parameter (w) High - Low | 0.091 | 0.428 |  |
| Risk sensitivity (μ)  High Stake Trial ( n = 78 ) | Weighting parameter (w) -Low Stake trial | -0.209 | 0.066 |  |
|  | Weighting parameter (w) High Stake trial | -.280* | 0.013 |  |
|  | Weighting parameter (w) High - Low | -0.02 | 0.859 |  |
| Risk sensitivity (μ)  High - Low ( n = 78 ) | Weighting parameter (w) -Low Stake trial | -0.163 | 0.154 |  |
|  | Weighting parameter (w) High Stake trial | -.245* | 0.031 |  |
|  | Weighting parameter (w) High - Low | -0.051 | 0.658 |  |
| PHQ (n =76) | Weighting parameter (w) Low reward trial | 0.073 | 0.532 |  |
|  | Weighting parameter (w) High reward trial | -0.070 | 0.549 |  |
|  | Weighting parameter (w) High - Low | -0.090 | 0.437 |  |
| PHQ (n =73) | Risk sensitivity (μ) - Low Stake Trial | 0.075 | 0.530 |  |
|  | Risk sensitivity (μ) - High Stake Trial | -0.062 | 0.601 |  |
|  | Risk sensitivity (μ) - High - Low | -0.084 | 0.479 |  |
|  |  |  |  |  |
| **Alcohol Users** | | Z (Coefficient) | P |  |
| AUDIT (n =40) | Weighting parameter (w) Low reward trial | -0.104 | 0.522 |  |
|  | Weighting parameter (w) High reward trial | -0.116 | 0.474 |  |
|  | Weighting parameter (w) High - Low | -0.012 | 0.943 |  |
| AUDIT (n =39) | Risk sensitivity (μ) - Low Stake Trial | -0.100 | 0.545 |  |
|  | Risk sensitivity (μ) - High Stake Trial | 0.104 | 0.528 |  |
|  | Risk sensitivity (μ) - High - Low | 0.026 | 0.875 |  |

**Table S2:** Correlation Analyses between Model-based vs -free weighting parameters, risk sensitivity parameters, AUDIT scores, and PHQ-9 scores in the total sample and alcohol user group.

## Correlation Analyses – With age as covariate

Correlational analyses revealed that for High stakes condition, the weighting parameter (ω) was negatively correlated with risk sensitivity $(\mu)$ (*r* = -.275, *p* = .018) across both users, less model-free control was associated with greater risk aversive behavior regardless of age.

- 1. **Cross-validation of RL Model and Utility Function**

To ensure that the observed correlation accurately reflects the associations between the model-based/model-free weighting (ω) and risk sensitivity (μ) parameters, we conducted a cross-validation procedure.

**Approach I**

Out of the 200 trials, we split our data into first and second 100 trials creating two datasets of 100 trials each per subject. This approach allowed us to preserve temporality and the change in the learning and risk sensitivity components of the decision-making process across the trials from immediately preceding actions.

The RL Model (M1) was used to estimate the model-based weighting parameter (ω) from the first 100 trials (A1) of the data, while the Utility Function (M2) was used to estimate the risk sensitivity parameter (μ) from the second 100 trials (A2). We then compared the resulting estimates (ω and μ) between M1A1 and M2A2. We then reversed the procedure by applying M2 to the first 100 trials (A1) and M1 to the second 100 trials (A2), and then compared the resulting parameters between M2A1 and M1A2. Since high-stakes trials showed significant correlations in the complete 200 trials, this analysis was performed on high-stakes trials only.

**Results**

In Alcohol Users, cross-validation correlation results in high stakes condition between model-based weighting parameter (ω) from M1A1 and the risk sensitivity parameter (μ) from M2A2 (r = -0.093, p = 0.588) and between ω from M1A2 and μ from M2A1 (r = -0.034, p = 0.843) were not statistically significant. These results are in stark contrasts with those observed when both models were applied to the entire data for each subject, which showed that ω was significantly negatively correlated with μ for high stakes condition in Alcohol Users, but not in Alcohol Non-users. Discordance between these results may stem from either A1 and A2 not having enough trials to reliably compute the two parameters, or there indeed is no association between the two and the significant association observed in the original analyses emerged because both parameters are estimated from the same dataset. However, since participants continue to learn task-contingencies throughout the task, it is presumable that ω from one half of the data may not be relevant to or correlated with μ from the second half.

**Approach II**

This involved splitting our data into odd and even trials, creating two datasets of 100 trials each per subject. This approach allowed us to preserve the internal consistency of the learning and risk sensitivity components of the decision-making process across the trials. Like the approach I, correlations were conducted between M1A2 and M2A1, and M1A1 and M2A2.

**Results**

In Alcohol Users, cross-validation yielded non-significant correlations in high stakes condition between ω from M1A1 and μ from M2A2 (r = -0.122, p = 0.479) and between ω from M1A2 and μ from M2A1 (r = -0.176, p = 0.297). As observed in results from approach I, possible explanation of the lack of significant correlation could be that either A1 and A2 do not have enough trials to reliably compute the model-based weighting parameter and risk sensitivity parameter, or there indeed is no association between the two and the significant association observed in the original analyses emerged because both parameters are estimated from the same datasets. Nevertheless, these findings need to be replicated with larger sample size with more robust cross-validation procedures.

1. **References**

1. d'Acremont, M., et al., *Neural correlates of risk prediction error during reinforcement learning in humans.* Neuroimage, 2009. **47**(4): p. 1929-39.

2. Bell, D.E., *Risk, Return, and Utility.* Management Science, 1995. **41**(January): p. 23-30.

3. Balasubramani, P.P., et al., *An extended reinforcement learning model of basal ganglia to understand the contributions of serotonin and dopamine in risk-based decision making, reward prediction, and punishment learning.* Front Comput Neurosci, 2014. **8**: p. 47.

4. Balasubramani, P.P., et al., *A network model of basal ganglia for understanding the roles of dopamine and serotonin in reward-punishment-risk based decision making.* Front Comput Neurosci, 2015. **9**: p. 76.

5. Mitchell, M., *An introduction to genetic algorithms*. Complex adaptive systems. 1996, Cambridge, Mass.: MIT Press. viii, 205 p.
